# Role of Entrepreneurial Behavior in Achieving Sustainable Digital Economy

**DOI:** 10.3389/fpubh.2022.829289

**Published:** 2022-02-25

**Authors:** Ying Wang, Han Zhou, Yanan Zhang, XiaoRan Sun

**Affiliations:** ^1^Collage of Economics and Management, Hebei Agricultural University, Baoding, China; ^2^Economics and Trade College, Hebei Finance University, Baoding, China; ^3^Research Office, Hebei Finance University, Baoding, China

**Keywords:** entrepreneurship, self-efficacy, digital economy, expectancy-value belief, entrepreneurial intention, entrepreneurial success

## Abstract

Entrepreneurship is a key indicator of not only personal growth but also economic growth by proposing a solution to worldwide problem of unemployment. The main purpose of this study is to measure the role of entrepreneurial self-efficacy and expectancy-value belief in digital economy among the students enrolled in universities of China. In this study quantitative approach was used for measuring the impact of entrepreneurial self-efficacy for achieving the entrepreneurial success along with expectancy-value belief wih the support of theories of entrepreneurial self-efficacy theory and achievement goal theory. The population frame for this study is the students of universities enrolled in degrees in China who were selected for data analysis through convenience sampling. The sample size for the study was 324. The data for the study has been analyzed using Smart-PLS software. The current study has been a contribution to the literature by measuring the role of entrepreneurial self-efficacy in entrepreneurial intentions, expectancy-value belief, and the entrepreneurial success and ultimate role in attaining the digital economy. The study has found that in this digital era, entrepreneurial success and expectancy-value belief are significantly predicted by entrepreneurial self-efficacy which in turn significantly predict the achievement of the digital economy in this progressing century. Furthermore, entrepreneurial success has been found to be an important mediator in the relationship between entrepreneurial self efficacy and digital economy for the present study. Moreover, entrepreneurial success has also significantly mediated the relationship of expectancy value belief and digital economy. This study is considerable for the universities in employing those study programs that help the motivated students in starting their businesses and such workshops should be made part of the curriculum to achieve their entrepreneurial goals and reach the expected success in entrepreneurship.

## Introduction

Entrepreneurial behavior can be influenced by the entrepreneur's aspirations instead of a set of inflexible strategic goals (it's possible that the entrepreneur's expectations are more realistic and ambitious than those of other firm managers). Entrepreneurial behavior is defined as identifying possibilities and putting good ideas into action. An individual or a group of people may carry out the succession of activities that this conduct necessitates, which normally demand ingenuity, determination, and personal initiative. Many people are born to be business owners. Behavior associated with entrepreneurship makes the major contribution to surviving external changes. It also has the decisive impact of achieving competitive advantages. Resultantly, encouraging entrepreneurial behavior has become a major aim in entrepreneurship, despite the fact that the method is still slightly elaborated through influences of the social institutions (1). Entrepreneurship is a struggle, wonderful experience, and success that requires specific attributes. It is said that human activity in venture conception, development, maintenance, and expansion should be studied in order to better understand entrepreneurial behavior (2). Studies of entrepreneurial behavior are focused on the tangible activities of individuals (single entrepreneurs or team members) at the beginning or early phases of a business. Entrepreneurial behavior is therefore the result of their desire, character, talents, expertise, knowledge, and talents, which are usually spread through action.

It is necessary to know what the digital economy is so a reference could be built up for entrepreneurial behaviors impacting the digital economy of any region. The digital economy is made up of a variety of general-purpose technologies as well as a wide range of social and economic activities carried out by individuals using the Web and associated technology. It includes routers, broadband lines, computers, cellular phones, Google and its apps, data analytics, cloud services, and their functionality, as well as the physical infrastructure on which digital technologies are built. There is no one-size-fits-all approach to entrepreneurship (3). The transformation of economy to the digital world is causing the emergence of a new kind of entrepreneurial behaviors based on aspects and characteristics that differ from the traditional rules of the game. These developments open up a slew of chances for businesses that can adapt to the new characteristics and functions associated with the spread of digital technology (4).

This article highlights key aspects that policymakers should take into account. Firstly, they have to provide the stimulus for the development of entrepreneurial activities in the digital sector. Secondly, those startups should be scaled up and the environment should be suitable for this purpose. These characteristics have features of the opposite nature to new initiatives' traditional flaws, which occur when they are unable to grow under more established societal or legal frameworks (5). When it comes to startups, the overriding focus on technological innovation might have an unclear consequence. Firms functioning in Western nations' so-called conventional industries (automobiles, home appliances, furnishings, etc.) frequently expand faster than those operating on the technology frontier. In this regard, the ability to develop new and more successful business models over time may be able to offset the technology factor's importance (6). Resultantly, not all companies which are achieving remarked growth are providing the remarked innovation in technology (7, 8).

To define entrepreneurial behaviors toward digital economy, it is mandatory to breakdown the behaviors into components which could lead to sustainable digital economies, such as self-efficacy, entrepreneurial intention, entrepreneurial success and expectancy value beliefs. The idea that one can attain particular goals is referred to as self-efficacy. Self-efficacy in entrepreneurship is utilized in entrepreneurial intention, and it refers to how confident entrepreneurs are regarding their abilities for the accomplishment of various projects and tasks (9). Business self-efficacy, a common trait among entrepreneurs, refers to their conviction in and attitude toward overcoming obstacles and achieving entrepreneurial success. Entrepreneurial self-efficacy has been shown in research to play a significant role in forecasting of entrepreneurial orientation and the encouragement of entrepreneurial performance (10).

According to the researchers, self-cognition of one's talents influences personal decisions, efforts, and behavior, implying that entrepreneurial self-efficacy could be a determinant of future actions (10). New entrants and the achievers in the field of entrepreneurship having stronger entrepreneurial self-efficacy tend to be having set targets and the goals related to their innovative businesses and they show more inclination toward engagement in the creative activities. It is also a fact that majority of entrepreneurs do possess confidence in themselves that they could achieve the targets they have set for themselves, but the reality is that the self efficacy associated with them is not passed on to the practices of entrepreneurial activity (11). After facing different risks associated with business, along with associated stress and tiredness emotionally, the majority of entrepreneurs are forced to resign (12).

In general, intentions are seen as the strongest indicator of action. Therefore the stronger the intention to perform the activity, the more likely the task will be completed. As a result, the great bulk of entrepreneurial behavior research over the last three decades has concentrated only on predicting and explaining what distinguishes people who express an ambition to start their own firm from those who do not (13). Such studies, however, constrain our present knowledge of entrepreneurial behaviors, as mounting evidence suggests that not all intentions are converted into real conduct when beginning and managing a new organization. Many people have the desire to start their own business, but owing to changes in their preferences or the advent of a new restriction, these desires are occasionally postponed or abandoned (14).

Several boundary circumstances do, in fact, impact the entrepreneurial intention-behavior relationship. Individual (e.g., age, gender, family business history) and contextual (e.g., uncertainty, avoidance and university atmosphere) characteristics affected the intention to behavior association (15). Similarly, It has been discovered that sex plays a role in the relationship of intention to behaviors. There is a scarcity of research on what success means to entrepreneurs (16). The most popular strategy for determining what success means to individual entrepreneurs has been to identify the most common factors that they use to define success, such as personal pleasure and money creation, and then quantify the variance in the value they place on these qualities (17). Dr. Martin Fishbein invented the expectancy-value theory in attempt to understand and anticipate an individual's attitude toward items and activities. The concept of expectation refers to the fact that most people will not choose to complete a task or continue to accomplish one if they expect to fail (15). The diverse views pupils have about the reasons they might participate in an activity are referred to “value.”

The three main components of the expectancy-value theory are belief, value, and expectations. Individuals first create beliefs about an object or action after receiving information about it. If the belief already exists, fresh knowledge may alter it. Individuals then assign a value to each quality that underpins a belief. Finally, depending on the computation of beliefs and values, an expectation is produced or adjusted (18). In connection to this theory, a connecting link of expectancy value beliefs could be developed between entrepreneurial intentions, entrepreneurial success, and a sustainable digital economy. There has been significant reported contribution of the variables mentioned and introduced above in different aspects of the researches. Our research focused on several objectives of achieving a sustainable digital economy. These variables had great connection among themselves in nourishing the old schools of economy and could be exploited well for devising a strong pavement for achieving sustainable digital economy so following objectives were formulated and tested for their significance in their contribution.

- To explore the relationship between entrepreneurial self efficacy and (entrepreneurial intention, entrepreneurial success, and expectancy value belief).
- To estimate the relationship between expectancy value belief and (entrepreneurial intention and entrepreneurial success).
- To identify the connection between entrepreneurial intention and (entrepreneurial success and digital economy).
- To determine relationship of entrepreneurial success with digital economy.
- To assess the mediating links of entrepreneurial intention and entrepreneurial success.

## Theoretical Support

### Self Efficacy Theory of Entrepreneurship

Self-efficacy is an individual's conviction in their capacity to do a specific activity. It is generally assumed that self-efficacy leads to the development of intentions. If a person owes feelings to the achievements of targets and goals, then there are strong chances that desire will be created for achieving them (19). Some people don't have the potential to achieve certain goals, so they will not make plans to pursue that objective. Individuals gain self-efficacy throughout time as they acquire a range of talents, such as social, cognitive, physical, and linguistic abilities, via life experiences (20). Past accomplishments, such as mastery of a task, boost self-efficacy, resulting in more ambitious intentions, or higher expectations. Self-efficacy may also be achieved by attentive observation of others' activities (social learning or vicarious learning), positive feedback, and self-reflection. Thus, if a person does well in a task when compared to comparable individuals they witness and is informed that they are performing well by others, they may conclude that they have the ability to pursue the next, more difficult work. According to self-efficacy theory, entrepreneurs will only undertake an entrepreneurial endeavor if they think they have the skills and ability to meet the obstacles presented by a given opportunity. If the challenge is too great for the potential entrepreneur, he or she may examine alternative possibilities, such as paid work. Individuals who believe their parents were high-performers seem more likely to believe they would start a business themselves, in comparison to people who have feelings about their parents that they could not achieve more in their lives. Children of entrepreneurs also have the feeling of achieving the set goals of establishing the startups as they have more expertise (21).

### Achievement Goal Theory

According to Murphy and Alexander, the field of research with the most categories and subcategories is based on the setting of goals and orientations for the goals (22). They accurately pointed toward comparable constructions that have been labeled under a variety of names. Simultaneously, there are some slight, but still significant, theoretical distinctions between different sorts of objectives that must be communicated through the use of different terminology. There appear to be three broad viewpoints on goals in existing literature in the context of accomplishment, each of quite a diverse nature. The social cognition study on aspirations of the people regarding targets or concerns is at quite oriented and directed (22). These can also be called as target goals (23). These target objectives do describe the standards or criteria by which individuals might evaluate their performance, but they don't truly address the reasons or motivations for which people would want to reach these targets (24).

A second level of objectives, on the other hand, is concerned with the more broad sorts of targets set by the people aiming at them along with finding the reasons for their setting (25). This goal content strategy tries to narrow down the number of objectives that might be used to support motivated action. Goals from a different viewpoint, such as achievement objectives, offer a middle ground between extremely detailed set targets and the constitution of the targets. Achievement objectives refers to the reasons and purposes for which a person is pursuing an achievement task. They are most commonly operationalized in terms of academic learning activities, but they may also be applied to other contexts of achievement such as athletic or corporate settings. Although task-specific goals and the more general goal content approach may be used to describe a variety of settings and types of goals (e.g., pleasure, safety), achievement goal structures were created expressly to explain accomplishment motivation and behavior.

Within achievement goal theory research, many labels and names have been employed for comparable goals. For example, the phrases “task-involved,” “learning,” “mastery objectives,” and “task,” are identified as goals developing and orientation of the people to focuss on the associated task to achieve mastery in it or know the procedures for doing it. Objectives which are concerned with people as how they focus on themselves along with their abilities and their performance sense in comparison to other people could be regarded as relative ability in terms of ego centric targets. A general response in several models and their outcomes is more oriented toward mastery and less orientation is observed toward adaptability during functionality in terms of how these goals are linked to various outcomes such as attributions, interest, affect, self-efficacy, persistence, self-regulation, levels of cognitive engagement, and choice behaviors. For these substantial discrepancies concerning goal stability causal relationships among crucial target orientations, several reasons of scientific nature are involved.

Further theory development and conduction of researches could be based on differentiation in terms of genuineness supporting empirical data. But that should not allow use of these terms haphazardly (26). This theory also provides the basis for identifying the mediating role of motivational factors for the attainment of digital economy in the larger perspective (27). So, entrepreneurial intention and the entrepreneurial success were used as the motivator variables in this research as mediators.

## Review of Literature and Hypothesis Development

### Relationship Between Entrepreneurial Self Efficacy and (Entrepreneurial Intention, Entrepreneurial Success, and Expectancy Value Belief)

Self-efficacy could be regarded as a variable due to its significance identified and defined through a theory of social cognition. It is described as a person's conviction in his or her own capacity to plan and carry out actions to accomplish the intended outcomes. This concept has gotten a lot of attention from educational scholars (19). Prior research has shown that performance-based results could be predicted through self-efficacy. Self-efficacy, for example, is thought to predict student academic success across all academic disciplines and levels. Despite the fact that directed effects of beliefs in self-efficacy on academic achievements are well established, very little research has been carried out to identify the mechanism which is motivationally attached to it and has a mediating role of it in the relationships of the achievements of targets (28). These studies are needed to understand how and why self-efficacy affects students' academic achievement and to design instructional actions and programs to improve academic achievement.

This kind of research is required to develop an understanding of the workings behind academic achievements of the scholars. It is also necessary to initiate the designs and models for the improvement of these kinds of relationships. The social cognitive Expectancy Value Model of Achievement Motivation (EVMAM), developed by Eccles and her colleagues based on Atkinson's expectancy-value model, is one of the most robust theories that integrates these components (29). Talking about the self efficacy levels of the people involved in the commerce field, comparisons were drawn between the two in the past proving that entrepreneurs had more self efficacy than the people who were not entrepreneurs, and this resulted in the understanding that self efficacy of the entrepreneurs could be utilized as a special characteristic in this type of research.

Self-efficacy has been shown to have a positive influence on faculty members' performance at Jordanian institutions, as well as a major role on faculty members of this sector regarding the process of teaching. However, it should not be the only thing in the direction of performance, but it has proved its worth. It is worth noting that among the four characteristics of self-efficacy, prior experience had the most impact (30). A study was conducted to evaluate the moderating and mediating roles of self-efficacy, and it was indicated that significance was achieved for its roles in the intention of entrepreneurship and personality. It was clearly indicated in the research that self-efficacy had a positive and strong role in terms of mediation and moderation between the already mentioned variables in that study.

The significance of self-efficacy in terms of behavior and performance was indicated during the exploration of the moderation between improvisations and the performance of the new startups and proved to be a contributing factor. Along with this, a negative association was found among improvisations and new startups of the innovators having self efficacy at lower levels (31). Entrepreneurial self-efficacy is an explanatory variable that distinguishes entrepreneurs from others by determining the potency of entrepreneurial intention and the likelihood of this intention leading to entrepreneurial behavior (32). According to a recent study, perceived self-efficacy influences feasibility and, in turn, leads to intention (30).

According to studies, having a high level of entrepreneurial self-efficacy enhances the likelihood of starting a new business. For example, research of undergraduate students revealed a positive and substantial association between entrepreneurial self-efficacy perception and entrepreneurial abilities, which included marketing, innovation, management, and financial control, risk taking, and entrepreneurial purpose. According to the study, those with more self-efficacy rated entrepreneurial prospects more favorably and were more likely to see good results (32). Self-efficacy is a psychological notion that motivates people to take action. It has been linked to goal selection, persistence, and performance in a variety of settings. This construct has been thoroughly researched and is clearly documented in the literature as having a considerable impact on performance in a variety of professions (33).

Resultantly, entrepreneurial self-efficacy is a larger idea of self-efficacy, which is a component of social cognitive theory that cannot be separated. Meanwhile, researchers defined entrepreneurial self-efficacy as an individual's conviction in their ability to be successful in carrying out company operations from numerous perspectives. Others, on the other hand, interpreted the concept as one's belief in one's potential to succeed in the process of beginning a business. Based on past solid and extensive research and meta-analyses, a theoretical framework relating self-efficacy, performance, and entrepreneurial success has been presented (34). As a result, multiple studies have found a link between self-efficacy and company performance, as assessed by income and/or workforce expansion, life satisfaction, and subjective pleasure (35).

According to the findings, task value was predicted by self-efficacy, and not in the reverse order. Previous research has found that not only two dimensions have a positive connection, but that task value is directly predicted by self-efficacy. In a previous study, self-efficacy was also shown to have large and direct influence on expectancies. Pupils with high self-efficacy have higher academic expectations and do better academically than students with low self-efficacy. These findings support Bandura's theory that antecedents of anticipation have self-efficacy because the outcomes of people's expectations are based mostly on their assessments of how well they would perform in a particular setting. Consequently, it is argued that self-efficacy promotes predicted behavior results causally, but not the other way around. In summary, the literature supported that there was a connection between expectancy value and self-efficacy and a relationship was found to be significant between beliefs of competence and task values (27). Based on this literature and the shortcomings of the previous researches, following hypotheses were formulated in this regard.

***H***_**1**_***:*** *Entrepreneurial self-efficacy plays a role in entrepreneurial intention*.
***H***_**2**_***:*** *Entrepreneurial self-efficacy plays a role in expectancy value belief*.
***H***_**3**_***:*** *Entrepreneurial self-efficacy plays a role in entrepreneurial success*.

### Relationship Between Expectancy Value Belief and (Entrepreneurial Intention and Entrepreneurial Success)

The Expectancy-Value Theory is based on a social cognitive approach to motivation. Individuals' choice, vigor, and persistence in performance can be anticipated and clarified mainly by their anticipations of the task value attribution and achievement, according to this school of thought; that really is, by their belief systems at how well those who will do the task and the extent to which they value the task. Aside from the above-mentioned elements, a few researchers of this field have incorporated another aspect related to sentiments. This final component is known as process expectancy. We defined process expectation in this case as the favorable sensations that someone anticipates having during their interactions. Indeed, no one starts anything that isn't useful or when the chances of success are slim, because finishing the activity in those sorts of situations is regarded as time wastage. Resultantly, nothing is initiated unless an anticipation of feeling good during the process is present. Consequently, these three ideas are regarded as key predictors of motivation (36). Four task-value components are identified by the current Expectancy-value theory.

However, it is acknowledged that interest in the subject is developed by the benefits associated with the scholars and the efforts and time of them. In a nutshell, the subject value components used in this type of work pertain to: (a) the significance of the topics and (b) the predicted cost-benefit relationship for passing the course. Expectancy value beliefs are developed prior to the start of class, based on past experiences, or they may have developed during the first few periods of time, during which the teacher student is learning about the curriculum, assessment, teaching approach, and so on. Keeping an eye on the expectancy value belief and its role in motivational aspects of the relationship, a very few studies have been carried out in past but a study based on expectancy value theory suggests its role for future research regarding self-efficacy and motivating factors such as entrepreneurial intention and entrepreneurial success (27). This showed a potential for analyzing the relationship among these, so we devised the following hypothesis for testing the significance.

***H***_**4**_***:*** *Expectancy value belief plays a role in entrepreneurial intention*.
***H***_**5**_***:*** *Expectancy value belief plays a role in entrepreneurial success*.

### Relationship of Entrepreneurial Intention and (Entrepreneurial Success and Digital Economy)

Establishment of the fact that entrepreneurial success is the outcome of entrepreneurial behavior toward achieving set goals plays an important role in determining the relationship between entrepreneurial intention and entrepreneurial success. Entrepreneurial behavior has been the subject of much investigation. Entrepreneurial intent is defined as a person's readiness to learn about entrepreneurship and commit to launching a new firm (37). Individuals' readiness to engage in entrepreneurial conduct or commitment to launching a new enterprise is defined as entrepreneurial ambition. This idea indicated that intention of the entrepreneurs had a significant and important impact on the conduction of the entrepreneurship startup, and it has been validated by studies (38). Entrepreneurial intention models have been shown to be an excellent indicator of gauging entrepreneurial behavior in previous studies, indicating their usefulness in understanding the entrepreneurial phenomenon.

Entrepreneurial purpose gives a person power and inspires them to engage in entrepreneurial conduct. It also shows how much work they are willing to put into business growth (15). Nevertheless, since many researchers have examined the link between an individual's entrepreneurial intentions and their successful entrepreneurial behavior patterns, the impact of entrepreneurial behavior, entrepreneurial alertness, entrepreneurial self-efficacy, and a proactive personality on entrepreneurial intentions and behaviors has yet to be thoroughly investigated. Existing research makes a major and beneficial addition to the relationship between entrepreneurial intention and the formulation of entrepreneurial conduct in the pursuit of entrepreneurial success (39). The use of digital technology, the internet, cellphones, and other information-gathering, storage, analysis, and sharing applications and technology are transforming the global economy, particularly through altering the entrepreneurial process. Thanks to digital technology, entrepreneurs may now start firms and offer their products and services all over the world. They're also affecting entrepreneurial intent, which is described as “an individual's personal conviction to take one or more specified activities in the process of seizing a new business opportunity. These relationships allowed us to devise certain hypotheses regarding the entrepreneurial intentions toward entrepreneurial success and digital economy.

- ***H***_**6**_***:*** *Entrepreneurial intention plays a role in entrepreneurial success*.
- ***H***_**7**_***:*** *Entrepreneurial intention plays a role in digital economy*.

### Relationship of Entrepreneurial Success With Digital Economy

The transformation of economies to the digital side is causing the emergence of a new dimension of entrepreneurship contributed by features that differ in traditional trends. Developments in this regard open up few chances for businesses that can adapt to the new characteristics and functions associated with the spread of digital technology. Entrepreneurship research has mostly concentrated on firm-level definitions of success and the human traits that help predict them, but it has not looked into what success means to entrepreneurs. When studying individual entrepreneurial success, the strategy has been to establish common success characteristics and assess the value of these to the entrepreneur (40).

Criteria-based methods, on the other hand, ignore the notion that entrepreneurs may attribute various interpretations to shared success criteria, which might affect how they build their businesses. The majority of studies indicate that entrepreneurs contribute significantly to the economy and society. However, understanding why some people fail to start a new business while others thrive is still insufficient. The process of starting a business has its own dynamics, order, and prerequisites. Various activities must be completed at distinct phases of the entrepreneurial process, and entrepreneurs must fulfill various responsibilities at each level (41). These processes lead to entrepreneurial success, which would certainly have an impact on achieving a digital economy, so we developed the following hypothesis.

***H***_**8**_***:*** *Entrepreneurial success plays a role in digital economy*.

### Mediating Role of Entrepreneurial Intention and Entrepreneurial Success

Various studies have been conducted in the past in which several factors like self-efficacy were used as mediators between entrepreneurial intentions, entrepreneurial success, and other variables, but no certain research has yet been conducted in identifying their roles as mediators among other variables. Some researchers have suggested using them as mediators to check the mediating role of both in achieving behaviors useful for the entrepreneurship. Such a research was carried out in past in which social media was used as a mediator between entrepreneurial intentions and the digital startups, which suggests its connection with these variables. So, we utilized this to use entrepreneurial intention as a mediator between entrepreneurial self efficacy and digital economy (42). Similarly, similar approach was used by Gali et al. (43) which identified the mediating role of social performance between entrepreneurial orientation and company success (43). This also suggested that entrepreneurial success could be utilized as mediator itself between our studied variables of self-efficacy and digital economy. Resultantly, it led us to develop the following hypotheses.

***H***_**9**_***:*** *Entrepreneurial intention mediates the relationship of entrepreneurial self efficacy and digital economy*.
***H***_**10**_***:*** *Entrepreneurial success mediates the relationship of entrepreneurial self efficacy and digital economy*.

**Figure 1:**
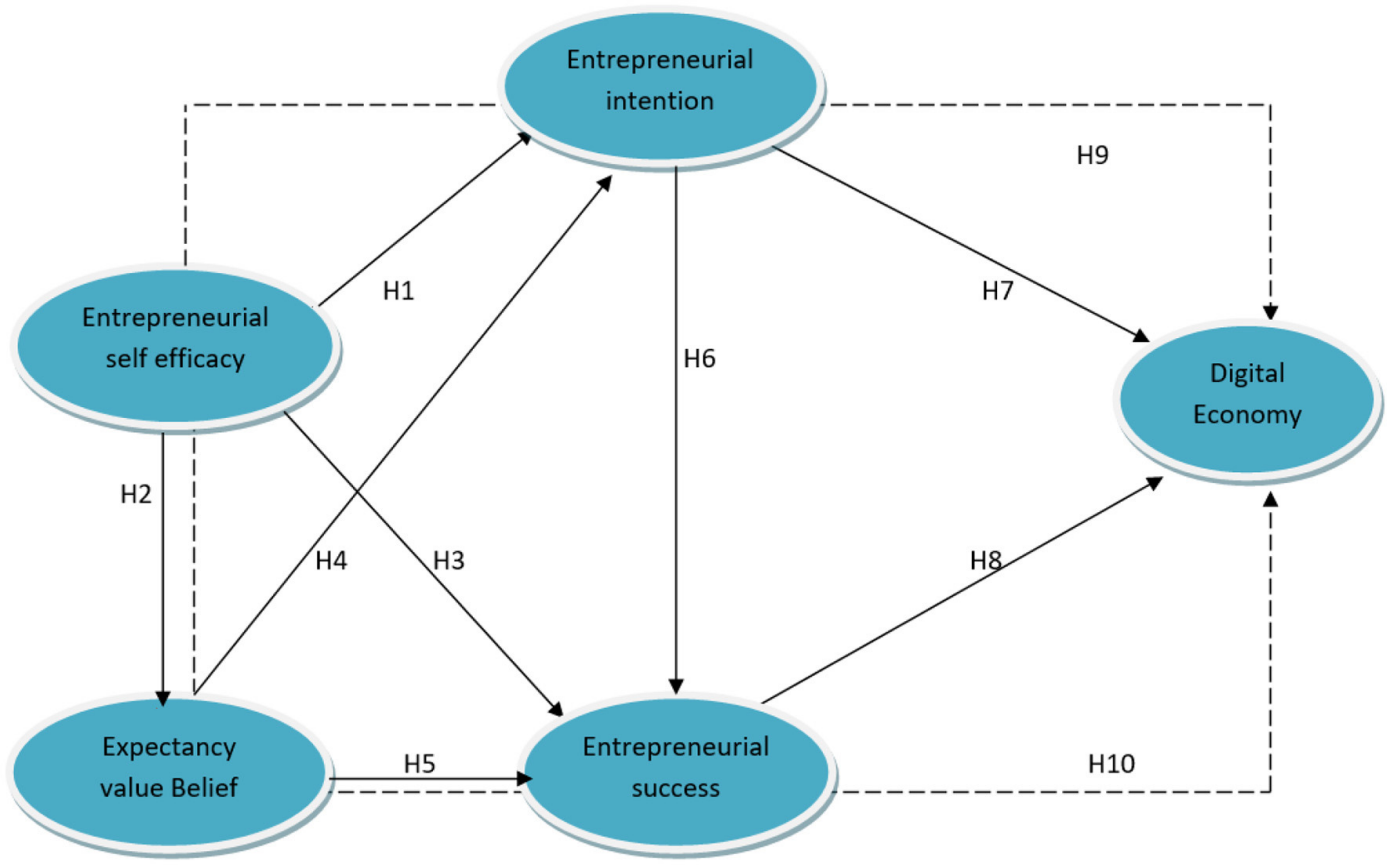
Theorethical framework.

Based on the above literature and hypothesis development, following therotical framework (see Figure 1) is formed.

## Research Methodology

In this study, quantitative methods have been used to analyze the data. The effect of some variables is checked on other variables; hence the positivist deductive approach of the study is used to minimize the biases in the study. A self-administered survey method has been used in this quantitative study for data collection. The population in the current study has been the students enrolled in the degree programs in China. The sample had been selected based on convenience sampling, as getting data of all the students in China is not possible; therefore, the students were reached according to the convenience and prior consent for their availability to get the responses with their free will. Therefore, the sample size of 324 has been selected based on convenience sampling. The unit of analysis for the present study is the individual's i.e., students and the usable questionnaires were received and used in the data analysis for this study. The study used structural equation modeling (SEM) via software Smart-PLS 3 for the measurement of hypothesis.

### Instrument Development

Data collection was done through surveys and the instrument used in the study was a questionnaire. The possible responses of the questionnaire were designed using a five-point Likert scale with 1 as strongly disagrees and five as strongly agree. The questionnaire was developed from previously used scales for the variables. The details for each variable have been given below.

#### Entrepreneurial Self Efficacy

The scale of entrepreneurial self-efficacy has been adopted from (44). It consisted of four items and the items included (1) How confident they were in successfully identifying new business opportunities? (2) How confident they were in successfully creating new products? (3) How confident they were in successfully thinking creatively? and (4) How confident they were in successfully commercializing an idea or new development?

#### Expectancy Value Belief

Expectancy value belief has been measured in this study with the help of four items taken from the previous study (27). The items were (1) Does entrepreneurship has so much value for me? (2) Can I invest time and effort in making my new business successful? (3) Will I be successful in this new business? and (4) Will I feel satisfied doing this new business?

#### Entrepreneurial Intentions

For entrepreneurial intentions, the scale was taken from the previously used scale by (45) which consisted of four items and the sample items are (1) How interested they were in engaging in prototypical entrepreneurial activities (starting a business)? (2) How interested they were in acquiring a small business? (3) How interested they were in starting and building a high-growth business?, and (4) how interested they were in and acquiring and building a company into a high-growth business) in the next 5–10 years?

#### Entrepreneurial Success

The items included in the measurement of entrepreneurial success were taken from (46). It included the items like (1) Firm performance is up to the expectations regarding market share, turnover, and profit growth, (2) Workplace relationships among the employees are strong, supportive, and satisfied, (3) Personal fulfillment has been achieved through work-life balance, own decision making and work flexibility, (4) Does your organization make an impact on the community through social responsibility, reputation, and environmentally friendly processes?, (5) Do your expected financial rewards are achieved?

#### Digital Economy

The variable of the digital economy is measured through seven items obtained from (4). And the questions were; (1) Digital adoption has affected the transportation, energy resources uses and urban living, (2) Digital adoption has affected the environmental aspect of life, (3) Digital adoption has affected educational, social and health perspectives of life, (4) Digital adoption has affected agriculture and rural development, (5) Digital adoption has affected business and industrial operations, (6) Digital adoption has affected finance and trade, (7) Digital adoption has affected the digital government systems and services.

### Demographic Analysis

The demographic diversity of the respondents had been checked through gender, age, education, and entrepreneurial status; the results have shown in Table 1. There were 185 females in the total respondents and 139 were males. While for age, 15–20 ages showed the frequency of 113, age 21–25 showed the frequency of 111, 26–30 showed the frequency of 74 and the lowest frequency was for the age segment of 31 years and above. Similarly, the highest frequency showed 142 among the bachelors, 129 were masters and 53 were Ph.D. however, for entrepreneurial status, 256 students showed their interest in the entrepreneurial activities, while 68 already owned business.

**Table 1:** Demographic frequencies and percentages.

**Demographics**	**Frequency**	**Percentage**
**Gender**		
Female	185	57.09%
Male	139	42.90%
**Age**		
15 to 20	113	34.87%
21 to 25	111	34.25%
26 to 30	74	22.83%
31 and above	26	8.02%
**Education**		
Bachelors	142	43.82%
Masters	129	39.81%
Ph.D. and others	53	16.35%
**Entrepreneurial Status**		
Intends to start a business	256	79.01%
Already own a business	68	20.09%

*N = 324*.

## Data Analysis

The data in the present study were analyzed through structural equation modeling, using the software Smart-PLS 3. In this software, the data is analyzed through the statistics obtained from the measurement model and the second stage shows the structural model for partial least square modeling (47). The data is declared significant measuring the validities and reliabilities while the hypotheses are measured with t-statistics and *p*-values.

### Measurement Model

**Figure 2:**
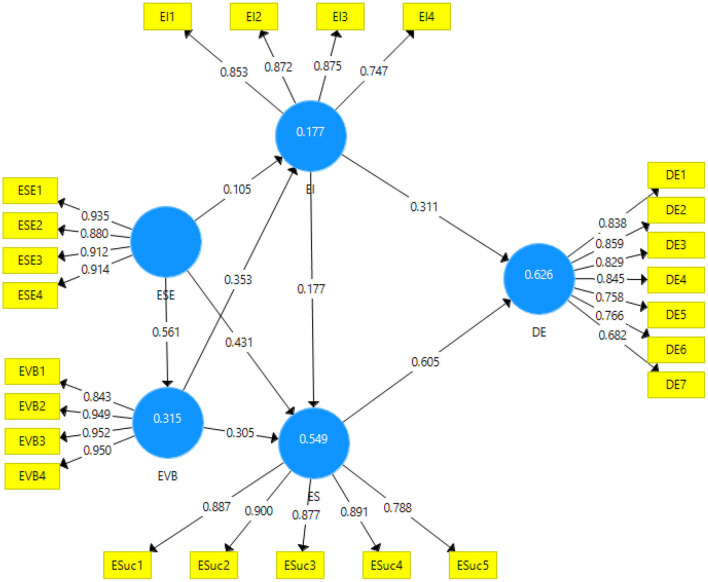
Measurement model algorithm. ESE, Entrepreneurial Self-Efficacy; EVB, Expected Value Belief; EI, Entrepreneurial intention; ES, Entrepreneurial success; DE, Digital Economy.

The data is validated in the measurement model; using the validities (factor loadings, AVE, Fronell, and Larcker criterion, HTMT ratio) and the reliabilities are checked with the help of Cronbach alpha reliability and Composite Reliability. The measurement model algorithm has been given in Figure 2.

The statistics obtained from the results for factor loadings, cronbach alpha, and composite reliability and average variance were extracted as shown in Table 2. The factor loadings of the items found for all variables have been found significant ranging from 0.747 to 0.950 which are among the good values where the value for acceptance is 0.7 while the composite reliability has also been found above the cut-off values (48). Moreover, the average variance extracted has also been among the accepTable value of being above 0.5 (49).

**Table 2:** Reliabilities and validities of the scale.

**Variables**	**Factor loadings**		**Cronbach alpha**	**Composite reliability**	**AVE**
Entrepreneurial self-efficacy	ESE1	0.935	**0.931**	**0.951**	**0.829**
	ESE 2	0.880			
	ESE 3	0.912			
	ESE 4	0.914			
Expected value belief	EVB1	0.843	**0.942**	**0.959**	**0.855**
	EVB 2	0.949			
	EVB 3	0.952			
	EVB 4	0.950			
Entrepreneurial intention	EI1	0.853	**0.859**	**0.904**	**0.703**
	EI 2	0.872			
	EI 3	0.875			
	EI 4	0.747			
Entrepreneurial success	ES1	0.887	**0.919**	**0.939**	**0.756**
	ES 2	0.900			
	ES 3	0.877			
	ES 4	0.891			
	ES 5	0.788			
Digital economy	DE1	0.838	**0.905**	**0.925**	**0.638**
	DE2	0.859			
	DE3	0.829			
	DE4	0.845			
	DE5	0.758			
	DE6	0.766			
	DE7	0.782			

*Bold values shows the significance between the variables they are commonly written bold*.

Furthermore, the r-square values of the dependent variables have also been found substantial. The r-square for the digital economy was the highest showing the contribution of 62.4% fitting the regression line. Secondly, entrepreneurial success showed a regression fit of 54.9% which is among very good fit for the model. Moving further to the dependent variables of expected value belief and entrepreneurial intention has shown the model fit of regression of 31.5 and 17.7% respectively.

The current study has used two techniques for checking the validities of the questionnaire. These two tests are Fornell and Larcker and HTMT ratios. The acceptance criteria for Fornell and Larcker criterion are that the highest value in each column should be the highest. This criterion is met in this study and the results for this can be seen in Table 3. The bold values verify the results.

**Table 3:** Fronell and larcker criteria.

	**DE**	**EI**	**ES**	**ESE**	**EVB**
DE	**0.799**				
EI	0.573	**0.838**			
ES	0.740	0.434	**0.870**		
ESE	0.580	0.303	0.656	**0.910**	
EVB	0.786	0.412	0.620	0.561	**0.925**

*ESE, Entrepreneurial Self-Efficacy; EVB, Expected Value Belief; EI, Entrepreneurial intentions; ES, Entrepreneurial success; DE, Digital Economy. Bold values shows the significance between the variables they are commonly written bold*.

Furthermore, the HTM ratios for the study have been obtained to show the validity of the measurement scales. For the HTMT ratio to be significant, these values must be below 0.9 (50). The results for the HTMT ratio are reported in Table 4.

**Table 4:** HTMT ratios.

	**DE**	**EI**	**ES**	**ESE**	**EVB**
DE					
EI	0.635				
ES	0.799	0.481			
ESE	0.629	0.329	0.708		
EVB	0.851	0.447	0.662	0.596	

*ESE, Entrepreneurial Self-Efficacy; EVB, Expected Value Belief; EI, Entrepreneurial intentions; ES, Entrepreneurial success; DE, Digital Economy*.

### Structural Model for the Measurement of Hypothesis

**Figure 3:**
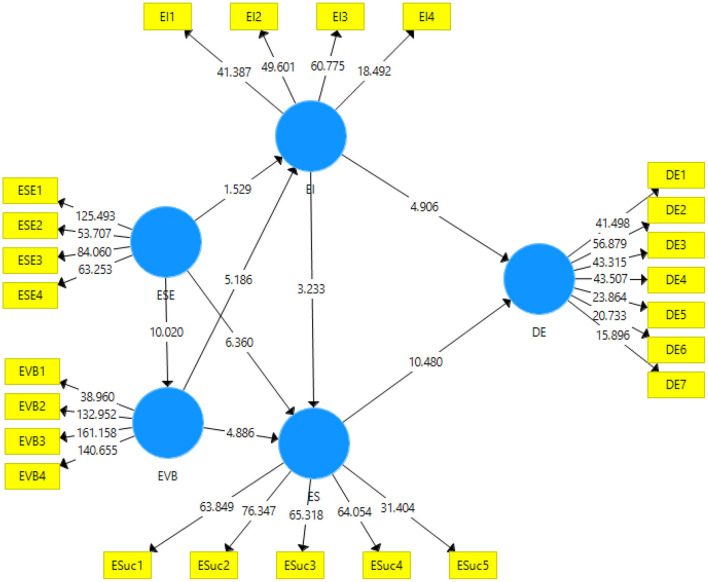
Measurement model algorithm. ESE, Entrepreneurial Self-Efficacy; EVB, Expected Value Belief; EI, Entrepreneurial intention; ES, Entrepreneurial success; DE, Digital Economy.

The hypothesis proposed in the study are either verified for rejection or acceptance based on the results obtained through the structural model algorithm of Smart-PLS. Original sample, mean values, standard deviation, t-statistics, and the *p*-values are the primary indicators for the acceptance or rejection of the hypothesis. The algorithm has been graphically represented in Figure 3 of the study obtained from the structural model.

The structural model algorithm gives the results for the direct effects and the indirect effects of the variables on other variables. These results are represented in separate tables. Following Tables 5, 6 give the direct and indirect effect of variables respectively.

**Table 5:** The direct effects of the variables.

**Paths**	**H**	**O**	**M**	**SD**	**T-Statistic**	***P*-value**	**Results**
ESE → EI	H_1_	0.105	0.106	0.068	1.529	0.127	Rejected
ESE → EVB	H_2_	0.561	0.561	0.056	10.020	0.000***	**Accepted**
ESE → ES	H_3_	0.431	0.428	0.068	6.360	0.000***	**Accepted**
EVB → EI	H_4_	0.353	0.357	0.068	5.186	0.000***	**Accepted**
EVB → ES	H_5_	0.305	0.308	0.062	4.886	0.000***	**Accepted**
EI → ES	H_6_	0.177	0.177	0.055	3.233	0.001**	**Accepted**
EI → DE	H_7_	0.311	0.313	0.063	4.906	0.000***	**Accepted**
ES → DE	H_8_	0.605	0.604	0.058	10.480	0.000***	**Accepted**

p*** < 0.001, p** < 0.00, H, Hypothesis; O, Original Sample; M, Sample Mean; SD, Standard Deviation; ESE, Entrepreneurial Self-Efficacy; EVB, Expected Value Belief; EI, Entrepreneurial intentions; ES, Entrepreneurial success; DE, Digital Economy. Bold values shows the significance between the variables they are commonly written bold.

**Table 6:** The direct effects of the variables.

**Paths**	**H**	**O**	**M**	**SD**	**T-Statistic**	***P*-value**	**Results**
ESE → EI → DE	H_9_	0.033	0.034	0.024	1.370	0.171	Rejected
ESE → ES → DE	H_10_	0.261	0.258	0.043	6.089	0.000***	***Accepted***

p*** < 0.001, H, Hypothesis; O, Original Sample; M, Sample Mean; SD, Standard Deviation; ESE, Entrepreneurial Self-Efficacy; EVB, Expected Value Belief; EI, Entrepreneurial intentions; ES, Entrepreneurial success; DE, Digital Economy. Bold values shows the significance between the variables they are commonly written bold.

In this study, the results show that the first hypothesis (H_1_: ESE → EI) has been rejected at *p* < 0.05 and the t-statistic = 1.529. However rest of the direct hypothesis have been accepted at different values of p in H_2_ (ESE → EVB), it is accepted at *p* < 0.001 and t-statistic = 10.02. Similarly, H_3_ (ESE → ES; *p* < 0.000 and t-statistic = 6.360), H_4_ (EVB → EI: *p* < 0.000 and t-statistic = 5.186), H_5_ (EVB → ES; *p* < 0.000 and t-statistic = 4.886) and H_6_ (EI → ES; *p* < 0.005 and t-statistic = 3.233) were accepted at their respective t-statistics and p-values. Furthermore the relationship of entrepreneurial intention and entrepreneurial success making H_7_ (EI → DE; *p* < 0.000 and t-statistic = 4.906) and H_8_ Es → DE; *p* < 0.000 and t-statistic = 10.480) have been approved with strong relationships.

The results for the mediating effects of entrepreneurial intention and entrepreneurial success have been reported in Table 6. The H_9_ indicating the hypothesis entrepreneurial intention mediates the relationship of entrepreneurial self-efficacy and the digital economy was rejected showing insignificant results. However, the H_10_ indicating that entrepreneurial success mediates the relationship of entrepreneurial self-efficacy and digital economy has been accepted with a *p* < 0.001 and t-statistic = 6.089.

## Discussion

This research focused on identifying the role of entrepreneurial behaviors in achieving a sustainable digital economy with certain objectives. The variables used in the study were derived from the research gaps and limitations of previous research by notable researchers. Main theme of the research was majorly based on entrepreneurial self-efficacy which has proven to have impact on successful entrepreneurship along with the intentions of entrepreneurship. Entrepreneurial intentions and entrepreneurial success were the derived outcomes of the entrepreneurial behaviors. In previously reported literature, it was identified that these behaviors could be utilized as the mediators in upcoming researches. To, check the combined effects of interconnected variables, this model was developed and tested. The results were quite interesting as out of ten developed hypotheses, eight were significant in their relationships. Only two were rejected due to their insignificance but we suggest that those should be tested in the future from different perspectives as they had strong potential. The reason for the rejection could be the inability of the respondents to understand their relationships properly.

The variables which were studied in our context were entrepreneurial self-efficacy, entrepreneurial intention, entrepreneurial success, expectancy value belief and the digital economy. Their direct effects, along with the mediations, were tested. Our first hypothesis was related to the analysis of the role of entrepreneurial self-efficacy toward entrepreneurial intentions, which was rejected due to its insignificance whereas, in past many researchers reported its significant impact on the entrepreneurial intentions such as (51). They reported that entrepreneurial self efficacy had strong impact on the entrepreneurial intention but that was in context to Turkish culture. Our second hypothesis was about checking the role of entrepreneurial self efficacy on expectancy value belief. Such kind of relationship for testing was suggested by (27). This proved its worth as this was accepted in our context of the study. Third hypothesis was about role of entrepreneurial self efficacy and entrepreneurial success. This kind of relation was never studied before but there was a scope of testing such relations (27). Results were significant in this regard which proved that self-efficacy in entrepreneurship could easily lead to the success in entrepreneurship and bringing in the innovation. Fourth and fifth hypothesis were about identifying the impact of expectancy value beliefs which were on the theoretical support of expectancy value theories.

Entrepreneurial intention proved to be the entrepreneurial behavior which had strong impact on achieving success in entrepreneurship and digital economy. Both of these hypotheses were accepted and these kind of relationships in our context of the research proved their significance of suggestions by (45) and (46). Lastly, entrepreneurial intention could not develop a significant point in determining their role as a mediator between entrepreneurial success and the digital economy as in past, its role was not studied as a mediator among any variable in any context. So, it provided a strong standpoint that it does not have a mediating role in any context so far but its role could be further explored using different variables. Entrepreneurial success proved its point as a mediator between entrepreneurial intention and the digital economy as success in entrepreneurship leads to be a helping hand between setting goals, intentions behind achieving those goals and the ultimate outcome of the digital economy. This kind of relationship was also suggested by (43). This research makes a significant contribution toward achieving a sustainable digital economy.

## Conclusion

Entrepreneurship is a primary key to personal and economic growth by finding a solution to the enormous problem of unemployment. From the supply chain perspective of China's structural reforms, entrepreneurship has the potential to promote and reform the industrial structure, economic transformation through high-tech procedures and techniques for achieving economic development through digitalization. The present study gives deep insights about the role of entrepreneurial self-efficacy in entrepreneurial success and expectancy-value belief which has been studied with the help of theories of entrepreneurial self-efficacy and achievement goal theory. The study has found that in this digital era, entrepreneurial success and expectancy-value belief are significantly predicted by entrepreneurial self-efficacy. Furthermore, entrepreneurial intentions, expectancy value belief and entrepreneurial success have been found to be the key drivers for digital economy by significantly predicting the achievement of the digital economy in this progressing century. However, the indirect effect measured in this study regarding the entrepreneurial intentions could not find any significance while the entrepreneurial success has been found to be an important mediator for the present study.

### Theoretical Contribution

The current study has been a contribution to the literature of entrepreneurship and organizational psychology by measuring the role of entrepreneurial self-efficacy in entrepreneurial intentions, expectancy-value belief, and entrepreneurial success and their ultimate role in attaining the digital economy. In this regard, the present study has provided evidence that entrepreneurial self-efficacy is an important constitutent of entrepreneurial success which ultimately boosts the digital economy of the country. Furthermore, it has also been established in this study that entrepreneurial intention is important among the people to digitalize their economy in a better way. Furthermore, another important contribution of the study is about stressing the role of expectancy value belief in this whole mechanism achieving digitalized economy through entreprenurship. Expectancy value belief has generously contributed toward mediating the relationship of entrepreneurial self efficacy and success. The literature of organizational psychology has also been upgraded with establishing a strong relationship of expectancy value belief with entrepreneurial intentions.

### Managerial Implications

This study is considerable for the universities in employing those study programs that help the motivated students in starting their businesses and such workshops should be made part of the curriculum to achieve their entrepreneurial goals and reach the expected success in entrepreneurship. Students should get engaged in the real life curricular activities that evokes the entrepreneurial skills in them by presenting them the real time scenarios. It is also important for the potential entrepreneurs that using hi-tech technologies in operations, contributes not only to their income but also plays a role in digitizing the economies at the mass level.

### Future Research and Limitations

However, there is a certain limitation to this study. First of all, this has used convenience sampling and this can be repeated in the future with probability sampling so the results could be validated. Secondly, it has used self-efficacy theory and achievement goal theory, however in the future it can be checked by incorporating the expectancy theory and other goal attainment theories considering the interpersonal and social systems. Further, this study should be conducted in other parts of the world to understand if there exists any cultural difference or how personality traits can affect this contribution of self-efficacy and entrepreneurship in the digital economy. Fourth, the model of the study can be repeated with additional variables to understand if there are some other attributes of individuals that may moderate these relationships.

## Data Availability Statement

The original contributions presented in the study are included in the article/supplementary material, further inquiries can be directed to the corresponding author.

## Ethics Statement

The studies involving human participants were reviewed and approved by Hebei Agricultural University (HAU), China. The patients/participants provided their written informed consent to participate in this study. The study was conducted in accordance with the Declaration of Helsinki.

## Author Contributions

YW conceived, designed, and wrote the article. YZ and XS help in data collection. HZ help in resources. All authors read and agreed to the published version of the manuscript.

## Funding

National Key R&D Program of China-Postharvest reducing losses, technology diffusion and comprehensive evaluation of wheat-corn in Hebei hydrothermal resource restricted area (Ref: 2018YFD0300507) and Hebei Social Science Fund Project-Research on measuring the poverty alleviation effect and promotion path of e-commerce in Hebei Province (Ref: HB20GL018).

## Conflict of Interest

The authors declare that the research was conducted in the absence of any commercial or financial relationships that could be construed as a potential conflict of interest.

## Publisher's Note

All claims expressed in this article are solely those of the authors and do not necessarily represent those of their affiliated organizations, or those of the publisher, the editors and the reviewers. Any product that may be evaluated in this article, or claim that may be made by its manufacturer, is not guaranteed or endorsed by the publisher.
